# Urban and air pollution: a multi-city study of long-term effects of urban landscape patterns on air quality trends

**DOI:** 10.1038/s41598-020-74524-9

**Published:** 2020-10-29

**Authors:** Lu Liang, Peng Gong

**Affiliations:** 1 Department of Geography and the Environment, University of North Texas, 1155 Union Circle, Denton, TX 76203 USA; 2 Ministry of Education Key Laboratory for Earth System Modeling, Department of Earth System Science, Tsinghua University, Beijing, China; 3 Tsinghua Urban Institute, Tsinghua University, Beijing, 100084 China; 4 Center for Healthy Cities, Institute for China Sustainable Urbanization, Tsinghua University, Beijing, 100084 China

**Keywords:** Environmental sciences, Environmental impact

## Abstract

Most air pollution research has focused on assessing the urban landscape effects of pollutants in megacities, little is known about their associations in small- to mid-sized cities. Considering that the biggest urban growth is projected to occur in these smaller-scale cities, this empirical study identifies the key urban form determinants of decadal-long fine particulate matter (PM_2.5_) trends in all 626 Chinese cities at the county level and above. As the first study of its kind, this study comprehensively examines the urban form effects on air quality in cities of different population sizes, at different development levels, and in different spatial-autocorrelation positions. Results demonstrate that the urban form evolution has long-term effects on PM_2.5_ level, but the dominant factors shift over the urbanization stages: area metrics play a role in PM_2.5_ trends of small-sized cities at the early urban development stage, whereas aggregation metrics determine such trends mostly in mid-sized cities. For large cities exhibiting a higher degree of urbanization, the spatial connectedness of urban patches is positively associated with long-term PM_2.5_ level increases. We suggest that, depending on the city’s developmental stage, different aspects of the urban form should be emphasized to achieve long-term clean air goals.

## Introduction

Air pollution represents a prominent threat to global society by causing cascading effects on individuals^1^, medical systems^2^, ecosystem health^3^, and economies^4^ in both developing and developed countries^5–8^. About 90% of global citizens lived in areas that exceed the safe level in the World Health Organization (WHO) air quality guidelines^9^. Among all types of ecosystems, urban produce roughly 78% of carbon emissions and substantial airborne pollutants that adversely affect over 50% of the world’s population living in them^5,10^. While air pollution affects all regions, there exhibits substantial regional variation in air pollution levels^11^. For instance, the annual mean concentration of fine particulate matter with an aerodynamic diameter of less than 2.5 $\upmu\mathrm{m}$ (PM_2.5_) in the most polluted cities is nearly 20 times higher than the cleanest city according to a survey of 499 global cities^12^. Many factors can influence the regional air quality, including emissions, meteorology, and physicochemical transformations. Another non-negligible driver is urbanization—a process that alters the size, structure, and growth of cities in response to the population explosion and further leads to lasting air quality challenges^13–15^.

With the global trend of urbanization^16^, the spatial composition, configuration, and density of urban land uses (refer to as urban form) will continue to evolve^13^. The investigation of urban form impacts on air quality has been emerging in both empirical^17^ and theoretical^18^ research. While the area and density of artificial surface areas have well documented positive relationship with air pollution^19–21^, the effects of urban fragmentation on air quality have been controversial. In theory, compact cities promote high residential density with mixed land uses and thus reduce auto dependence and increase the usage of public transit and walking^21,22^. The compact urban development has been proved effective in mitigating air pollution in some cities^23,24^. A survey of 83 global urban areas also found that those with highly contiguous built-up areas emitted less NO_2_^22^. In contrast, dispersed urban form can decentralize industrial polluters, improve fuel efficiency with less traffic congestion, and alleviate street canyon effects^25–28^. Polycentric and dispersed cities support the decentralization of jobs that lead to less pollution emission than compact and monocentric cities^29^. The more open spaces in a dispersed city support air dilution^30^. In contrast, compact cities are typically associated with stronger urban heat island effects^31^, which influence the availability and the advection of primary and secondary pollutants^32^.

The mixed evidence demonstrates the complex interplay between urban form and air pollution, which further implies that the inconsistent relationship may exist in cities at different urbanization levels and over different periods^33^. Few studies have attempted to investigate the urban form–air pollution relationship with cross-sectional and time series data^34–37^. Most studies were conducted in one city or metropolitan region^38,39^ or even at the country level^40^. Furthermore, large cities or metropolitan areas draw the most attention in relevant studies^5,41,42^, and the small- and mid-sized cities, especially those in developing countries, are heavily underemphasized. However, virtually all world population growth^43,44^ and most global economic growth^45,46^ are expected to occur in those cities over the next several decades. Thus, an overlooked yet essential task is to account for various levels of cities, ranging from large metropolitan areas to less extensive urban area, in the analysis.

This study aims to improve the understanding of how the urban form evolution explains the decadal-long changes of the annual mean PM_2.5_ concentrations in 626 cities at the county-level and above in China. China has undergone unprecedented urbanization over the past few decades and manifested a high degree of heterogeneity in urban development^47^. Thus, Chinese cities serve as a good model for addressing the following questions: (1) whether the changes in urban landscape patterns affect trends in PM_2.5_ levels? And (2) if so, do the determinants vary by cities?

## Data

### City boundaries

Our study period spans from the year 2000 to 2014 to keep the data completeness among all data sources. After excluding cities with invalid or missing PM_2.5_ or sociodemographic value, a total of 626 cities, with 278 prefecture-level cities and 348 county-level cities, were selected. City boundaries are primarily based on the Global Rural–Urban Mapping Project (GRUMP) urban extent polygons that were defined by the extent of the nighttime lights^48,49^. Few adjustments were made. First, in the GRUMP dataset, large agglomerations that include several cities were often described in one big polygon. We manually split those polygons into individual cities based on the China Administrative Regions GIS Data at 1:1 million scales^50^. Second, since the 1978 economic reforms, China has significantly restructured its urban administrative/spatial system. Noticeable changes are the abolishment of several prefectures and the promotion of many former county-level cities to prefecture-level cities^51^. Thus, all city names were cross-checked between the year 2000 and 2014, and the mismatched records were replaced with the latest names.

### PM_2.5_ concentration data

The annual mean PM_2.5_ surface concentration (micrograms per cubic meter) for each city over the study period was calculated from the Global Annual PM_2.5_ Grids at 0.01° resolution^52^. This data set combines Aerosol Optical Depth retrievals from multiple satellite instruments including the NASA Moderate Resolution Imaging Spectroradiometer (MODIS), Multi-angle Imaging SpectroRadiometer (MISR), and the Sea-Viewing Wide Field-of-View Sensor (SeaWiFS). The global 3-D chemical transport model GEOS-Chem is further applied to relate this total column measure of aerosol to near-surface PM_2.5_ concentration, and geographically weighted regression is finally used with global ground-based measurements to predict and adjust for the residual PM_2.5_ bias per grid cell in the initial satellite-derived values.

### Human settlement layer

The urban forms were quantified with the 40-year (1978–2017) record of annual impervious surface maps for both rural and urban areas in China^47,53^. This state-of-art product provides substantial spatial–temporal details on China’s human settlement changes. The annual impervious surface maps covering our study period were generated from 30-m resolution Landsat images acquired onboard Landsat 5, 7, and 8 using an automatic “Exclusion/Inclusion” mapping framework^54,55^. The output used here was the binary impervious surface mask, with the value of one indicating the presence of human settlement and the value of zero identifying non-residential areas. The product assessment concluded good performance. The cross-comparison against 2356 city or town locations in GeoNames proved an overall high agreement (88%) and approximately 80% agreement was achieved when compared against visually interpreted 650 urban extent areas in the year 1990, 2000, and 2010.

### Control variables

To provide a holistic assessment of the urban form effects, we included control variables that are regarded as important in influencing air quality to account for the confounding effects.

Four variables, separately population size, population density, and two economic measures, were acquired from the China City Statistical Yearbook^56^ (National Bureau of Statistics 2000–2014). Population size is used to control for the absolute level of pollution emissions^41^. Larger populations are associated with increased vehicle usage and vehicle-kilometers travels, and consequently boost tailpipes emissions^5^. Population density is a useful reflector of transportation demand and the fraction of emissions inhaled by people^57^. We also included gross regional product (GRP) and the proportion of GRP generated from the secondary sector (GRP2). The impact of economic development on air quality is significant but in a dynamic way^58^. The rising per capita income due to the concentration of manufacturing industrial activities can deteriorate air quality and vice versa if the stronger economy is the outcome of the concentration of less polluting high-tech industries. Meteorological conditions also have short- and long-term effects on the occurrence, transport, and dispersion of air pollutants^59–61^. Temperature affects chemical reactions and atmospheric turbulence that determine the formation and diffusion of particles^62^. Low air humidity can lead to the accumulation of air pollutants due to it is conducive to the adhesion of atmospheric particulate matter on water vapor^63^. Whereas high humidity can lead to wet deposition processes that can remove air pollutants by rainfall. Wind speed is a crucial indicator of atmospheric activity by greatly affect air pollutant transport and dispersion. All meteorological variables were calculated based on China 1 km raster layers of monthly relative humidity, temperature, and wind speed that are interpolated from over 800 ground monitoring stations^64^. Based on the monthly layer, we calculated the annual mean of each variable for each year. Finally, all pixels falling inside of the city boundary were averaged to represent the overall meteorological condition of each city.

## Methods

**Figure 1:**
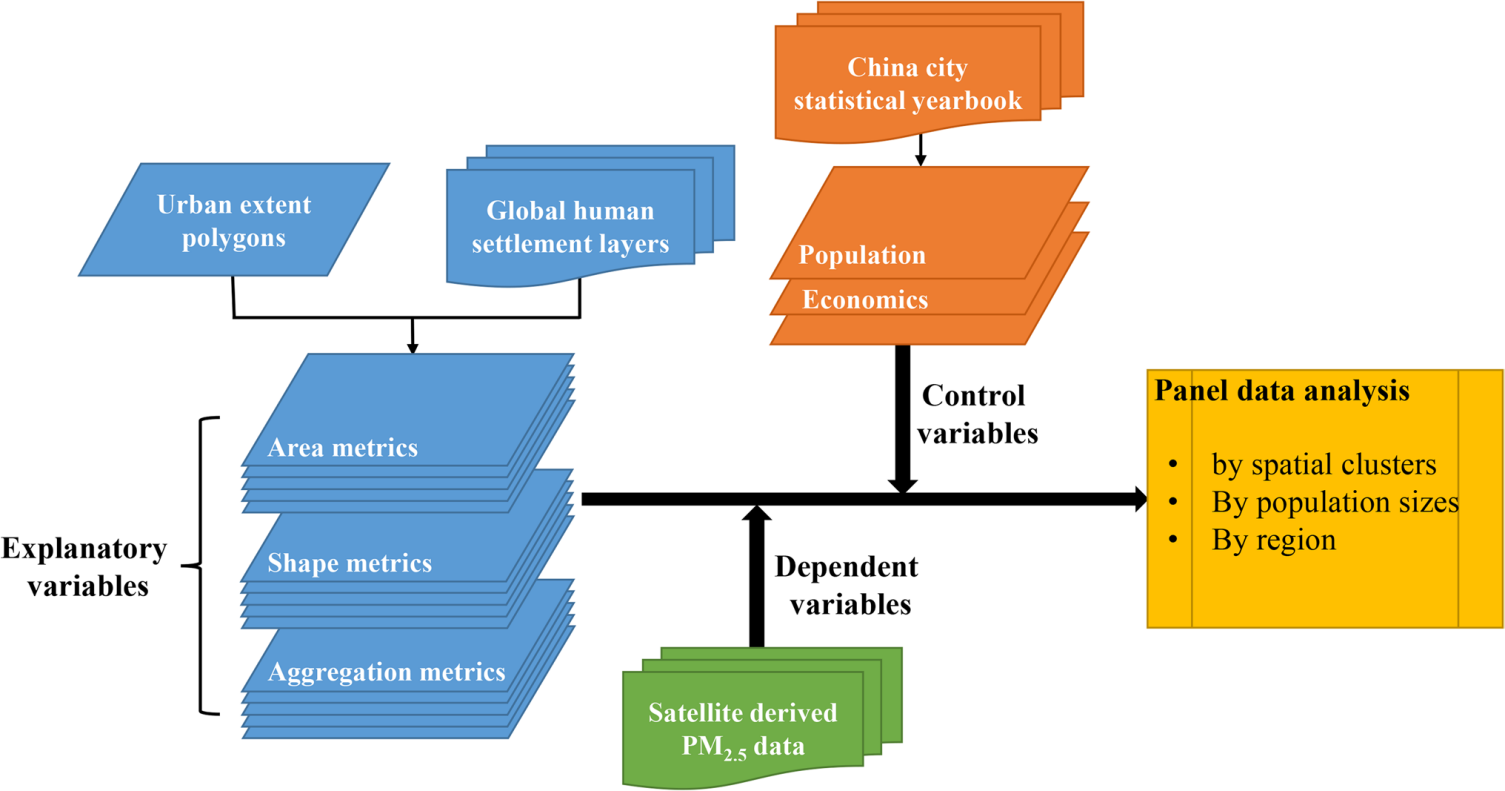
Methodology workflow.

Considering the dynamic urban form-air pollution relationship evidenced from the literature review, our hypothesis is: the determinants of PM_2.5_ level trends are not the same for cities undergoing different levels of development or in different geographic regions. To test this hypothesis, we first categorized city groups following (1) social-economic development level, (2) spatial autocorrelation relationship, and (3) population size. We then assessed the relationship between urban form and PM_2.5_ level trends by city groups. Finally, we applied the panel data models to different city groups for hypothesis testing and key determinant identification (Fig. 1).

### Calculation of urban form metrics

Based on the previous knowledge^65–67^, fifteen landscape metrics falling into three categories, separately area, shape, and aggregation, were selected. Those metrics quantify the compositional and configurational characteristics of the urban landscape, as represented by urban expansion, urban shape complexity, and compactness (Table 1).

**Table 1:** Definition and description of the urban form metrics.

	Variables	Acronym	Unit	Definition
Area metrics	Total area	TA	km^2^	The total area of the landscape
	Largest patch index	LPI	Percent	The area of the largest urban patch divided by total landscape area
	Number of patches	NP	None	Number of urban patches in the landscape
	Patch density	PD	Number per hectares	$\frac{n}{A}\times {10{,}000}$
	Mean patch size	AREA_MN	km^2^	The average size of all urban patches
Shape metrics	Mean patch shape index	SHAPE_MN	None	${\sum }_{j=1}^{n}\frac{0.25\times {P}_{j}}{\sqrt{{a}_{j}}}/n$. It increases as patch shape becomes more irregular
	Mean patch fractal dimension	FRAC_MN	None	${\sum }_{j=1}^{n}\frac{2\times \mathrm{ln}(0.25\times {P}_{j)}}{\mathrm{ln}({a}_{j})}/n$. FRAC increases with higher convoluted, plane-filling perimeters
	Mean contiguity index	CONTIG_MN	None	${\sum }_{j=1}^{n}\frac{\left[\frac{{\sum }_{r=1}^{z}{C}_{jz}}{{a}_{j}}\right]-1}{\mathrm{v}-1}/n$. CONTIG equals 0 for a one-pixel patch and increases to a limit of 1 as patch contiguity increases
Aggregation metrics	Mean nearest neighbor distance	ENN_MN	meter	ENN decreases as the distance to the nearest neighbor decreases
	Landscape shape index	LSI	None	$\frac{E}{\mathrm{min}E}.$ LSI increases as landscape shape becomes more irregular or as the length of edge within the landscape increases
	Patch cohesion index	COHESION	None	$\left[1-\frac{\sum_{j=1}^{n}{p}_{j}^{*}}{\sum_{j=1}^{n}{p}_{j}^{*}\sqrt{{a}_{j}^{*}}}\right]{[1-\frac{1}{\sqrt{Z}}]}^{-1}\times 100$. COHESION measures the physical connectedness of the urban patch
	Splitting index	SPLIT	None	$\frac{{A}^{2}}{\sum_{j=1}^{n}{a}_{ij}^{2}}$. SPLIT increases as the urban patches are subdivided into smaller patches and decreases in area
	Landscape division index	DIVISION	Proportion	$[1-\sum_{j=1}^{n}{(\frac{{a}_{j}}{A})}^{2})]$. DIVISION approaches one, as the proportion of the landscape comprised of the urban patches decreases and patch size decreases
	Effective mesh size	MESH	Hectares	$\frac{\sum_{j=1}^{n}{a}_{ij}^{2}}{A}(\frac{1}{\mathrm{10,000}})$. MESH reaches the maximum when the landscape consists of one single patch
	Aggregation index	AI	Percent	$\left(\frac{{g}_{ii}}{max\to {g}_{ii}}\right){P}_{i}\times 100$. AI increases as the landscape is increasingly aggregated
Control variables	Total population	POP	10,000	
		GDP	10,000 yuan	
		GDP2	10,000 yuan	

$\mathrm{A}$, total landscape area; ${a}_{j}$, area of patch $\mathrm{j}$; ${C}_{jr}$, contiguity value for pixel r in patch $\mathrm{j}$; $\mathrm{E}$, total length of the edge in landscape in terms of cell surfaces; ${g}_{ii}$, number of like adjacencies between pixels of urban patch $i$ based on the single-count method; ${max\to g}_{ii}$, maximum number of like adjacencies between pixels of urban patch;$\mathrm{n}$, number of urban patches; ${p}_{j}$, perimeter of patch $\mathrm{j}$;$\mathrm{v}$, sum of the values in a 3-by-3 cell template; . denotes *p* value < 0.1; *p value < 0.05; **p value < 0.01; ***p value < 0.001. Cells in grey shadow show descriptive statistics of the corresponding variables in 2014.

Area metrics gives an overview of the urban extent and the size of urban patches that are correlated with PM_2.5_^20^. As an indicator of the urbanization degree, total area (TA) typically increases constantly or remains stable, because the urbanization process is irreversible. Number of patches (NP) refers to the number of discrete parcels of urban settlement within a given urban extent and Mean Patch Size (AREA_MN) measures the average patch size. Patch density (PD) indicates the urbanization stages. It usually increases with urban diffusion until coalescence starts, after which decreases in number^66^. Largest Patch Index (LPI) measures the percentage of the landscape encompassed by the largest urban patch.

The shape complexity of urban patches was represented by Mean Patch Shape Index (SHAPE_MN), Mean Patch Fractal Dimension (FRAC_MN), and Mean Contiguity Index (CONTIG_MN). The greater irregularity the landscape shape, the larger the value of SHAPE_MN and FRAC_MN. CONTIG_MN is another method of assessing patch shape based on the spatial connectedness or contiguity of cells within a patch. Larger contiguous patches will result in larger CONTIG_MN.

Aggregation metrics measure the spatial compactness of urban land, which affects pollutant diffusion and dilution. Mean Euclidean nearest-neighbor distance (ENN_MN) quantifies the average distance between two patches within a landscape. It decreases as patches grow together and increases as the urban areas expand. Landscape Shape Index (LSI) indicates the divergence of the shape of a landscape patch that increases as the landscape becomes increasingly disaggregated^68^. Patch Cohesion Index (COHESION) is suggestive of the connectedness degree of patches^69^. Splitting Index (SPLIT) and Landscape Division Index (DIVISION) increase as the separation of urban patches rises, whereas, Mesh Size (MESH) decreases as the landscape becomes more fragmented. Aggregation Index (AI) measures the degree of aggregation or clumping of urban patches. Higher values of continuity indicate higher building densities, which may have a stronger effect on pollution diffusion.

The detailed descriptions of these indices are given by the FRAGSTATS user’s guide^70^. The calculation input is a layer of binary grids of urban/nonurban. The resulting output is a table containing one row for each city and multiple columns representing the individual metrics.

### Division of cities

#### Division based on the socioeconomic development level

The socioeconomic development level in China is uneven. The unequal development of the transportation system, descending in topography from the west to the east, combined with variations in the availability of natural and human resources and industrial infrastructure, has produced significantly wide gaps in the regional economies of China. By taking both the economic development level and natural geography into account, China can be loosely classified into Eastern, Central, and Western regions. Eastern China is generally wealthier than the interior, resulting from closeness to coastlines and the Open-Door Policy favoring coastal regions. Western China is historically behind in economic development because of its high elevation and rugged topography, which creates barriers in the transportation infrastructure construction and scarcity of arable lands. Central China, echoing its name, is in the process of economic development. This region neither benefited from geographic convenience to the coast nor benefited from any preferential policies, such as the Western Development Campaign.

#### Division based on spatial autocorrelation relationship

The second type of division follows the fact that adjacent cities are likely to form air pollution clusters due to the mixing and diluting nature of air pollutants^71^, i.e., cities share similar pollution levels as its neighbors. The underlying processes driving the formation of pollution hot spots and cold spots may differ. Thus, we further divided the city into groups based on the spatial clusters of PM_2.5_ level changes.

Local indicators of spatial autocorrelation (LISA) was used to determine the local patterns of PM_2.5_ distribution by clustering cities with a significant association. In the presence of global spatial autocorrelation, LISA indicates whether a variable exhibits significant spatial dependence and heterogeneity at a given scale^72^. Practically, LISA relates each observation to its neighbors and assigns a value of significance level and degree of spatial autocorrelation, which is calculated by the similarity in variable $z$ between observation $i$ and observation $j$ in the neighborhood of $i$ defined by a matrix of weights ${w}_{ij}$^7,73^:

$${I}_{i}=\frac{{z}_{i}-\bar{z}}{{\sigma }^{2}}\sum_{j=1,j\ne i}^{n}[{w}_{ij}({z}_{j}-\bar{z})]$$

where ${I}_{i}$ is the Moran’s I value for location $i$; ${\sigma }^{2}$ is the variance of variable $z$; $\bar{z}$ is the average value of $z$ with the sample number of $n$. The weight matrix ${w}_{ij}$ is defined by the k-nearest neighbors distance measure, i.e., each object’s neighborhood consists of four closest cites.

**Figure 2:**
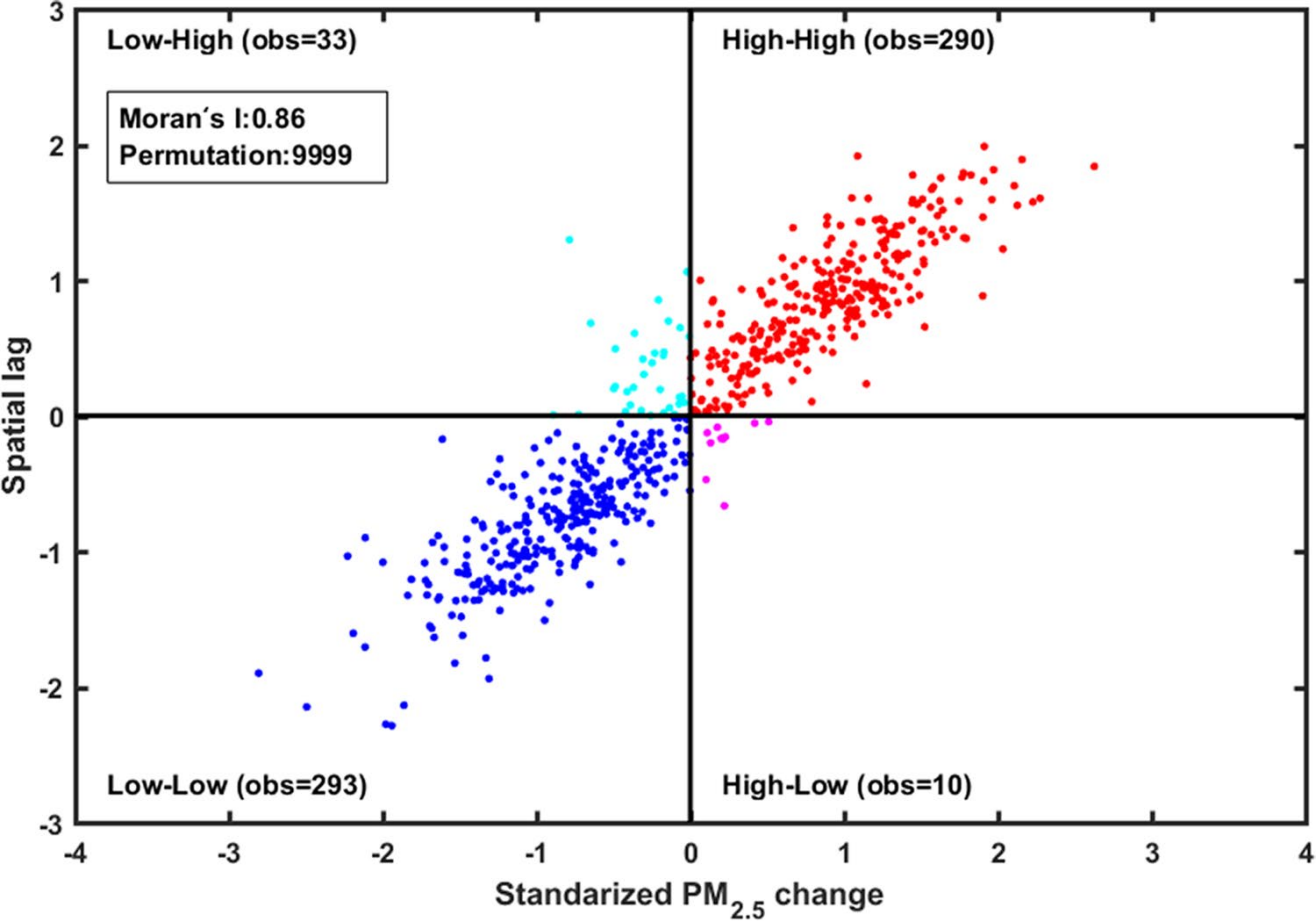
Moran’s I scatterplot. Figure was produced by R 3.4.3^76^.

The computation of Moran’s I enables the identification of hot spots and cold spots. The hot spots are high-high clusters where the increase in the PM_2.5_ level is higher than the surrounding areas, whereas cold spots are low-low clusters with the presence of low values in a low-value neighborhood. A Moran scatterplot, with x-axis as the original variable and y-axis as the spatially lagged variable, reflects the spatial association pattern. The slope of the linear fit to the scatter plot is an estimation of the global Moran's I^72^ (Fig. 2). The plot consists of four quadrants, each defining the relationship between an observation^74^. The upper right quadrant indicates hot spots and the lower left quadrant displays cold spots^75^.

#### Division based on population size

The last division was based on population size, which is a proven factor in changing per capita emissions in a wide selection of global cities, even outperformed land urbanization rate^77–79^. We used the 2014 urban population to classify the cities into four groups based on United Nations definitions^80^: (1) large agglomerations with a total population larger than 1 million; (2) mid-sized cities, 500,000–1 million; (3) small cities, 250,000–500,000, and (4) very small cities, 100,000–250,000.

### Panel data analysis

The panel data analysis is an analytical method that deals with observations from multiple entities over multiple periods. Its capacity in analyzing the characteristics and changes from both the time-series and cross-section dimensions of data surpasses conventional models that purely focus on one dimension^81,82^. The estimation equation for the panel data model in this study is given as:

$$ \begin{aligned} {\text{lnPM}}_{{{2}{\text{.5it}}}} & { = }\upbeta _{{0}} +\upbeta _{{1}} {\text{ ln TA}}_{{{\text{it}}}} { + }\upbeta _{{2}} {\text{ ln LPI}}_{{{\text{it}}}} { + }\upbeta _{{3}} {\text{ ln NP}}_{{{\text{it}}}} { + }\upbeta _{{4}} {\text{ ln PD}}_{{{\text{it}}}} { + }\upbeta _{{5}} {\text{ ln AREA}}\_{\text{MN}}_{{{\text{it}}}} \\ & \quad + \upbeta _{{6}} {\text{ ln SHAPE}}\_{\text{MN}}_{{{\text{it}}}} { + }\upbeta _{{7}} {\text{ ln FRAC}}\_{\text{MN}}_{{{\text{it}}}} { + }\upbeta _{{8}} {\text{ ln CONTIG}}\_{\text{MN}}_{{{\text{it}}}} { + }\upbeta _{{9}} {\text{ ln ENN}}\_{\text{MN}}_{{{\text{it}}}} \\ & \quad + \upbeta _{{{10}}} {\text{ ln LSI}}_{{{\text{it}}}} { + }\upbeta _{{{11}}} {\text{ ln COHESION}}_{{{\text{it}}}} { + }\upbeta _{{{12}}} {\text{ ln SPLIT}}_{{{\text{it}}}} { + }\upbeta _{{{13}}} {\text{ ln DIVISION}}_{{{\text{it}}}} { + }\upbeta _{{{14}}} {\text{ ln MESH}}_{{{\text{it}}}} \\ & \quad + \upbeta _{{{15}}} {\text{ ln AI}}_{{{\text{it}}}} { + }\upbeta _{{{16}}} {\text{ ln POP}}_{{{\text{it}}}} { + }\upbeta _{{{17}}} {\text{ ln GDP}}_{{{\text{it}}}} { + }\upbeta _{{{18}}} {\text{ ln GDP2}}_{{{\text{it}}}} + \varepsilon_{it} \\ \end{aligned} $$

where the subscript $i$ and $t$ refer to city and year respectively. $\upbeta _{{0}}$ is the intercept parameter and $\upbeta _{{1}} - { }\upbeta _{{{18}}}$ are the estimates of slope coefficients. $\varepsilon$ is the random error. All variables are transformed into natural logarithms.

Two methods can be used to obtain model estimates, separately fixed effects estimator and random effects estimator. The fixed effects estimator assumes that each subject has its specific characteristics due to inherent individual characteristic effects in the error term, thereby allowing differences to be intercepted between subjects. The random effects estimator assumes that the individual characteristic effect changes stochastically, and the differences in subjects are not fixed in time and are independent between subjects. To choose the right estimator, we run both models for each group of cities based on the Hausman specification test^83^. The null hypothesis is that random effects model yields consistent and efficient estimates^84^: ${H}_{0}{:}\,E\left({\varepsilon }_{i}|{X}_{it}\right)=0$. If the null hypothesis is rejected, the fixed effects model will be selected for further inferences. Once the better estimator was determined for each model, one optimal panel data model was fit to each city group of one division type. In total, six, four, and eight runs were conducted for socioeconomic, spatial autocorrelation, and population division separately and three, two, and four panel data models were finally selected.

## Results

### Spatial patterns of PM_2.5_ level changes

During the period from 2000 to 2014, the annual mean PM_2.5_ concentration of all cities increases from 27.78 to 42.34 µg/m^3^, both of which exceed the World Health Organization recommended annual mean standard (10 µg/m^3^). It is worth noting that the PM_2.5_ level in the year 2014 also exceeds China’s air quality Class 2 standard (35 µg/m^3^) that applies to non-national park places, including urban and industrial areas. The standard deviation of annual mean PM_2.5_ values for all cities increases from 12.34 to 16.71 µg/m^3^, which shows a higher variability of inter-urban PM_2.5_ pollution after a decadal period. The least and most heavily polluted cities in China are Delingha, Qinghai (3.01 µg/m^3^) and Jizhou, Hubei (64.15 µg/m^3^) in 2000 and Hami, Xinjiang (6.86 µg/m^3^) and Baoding, Hubei (86.72 µg/m^3^) in 2014.

**Figure 3:**
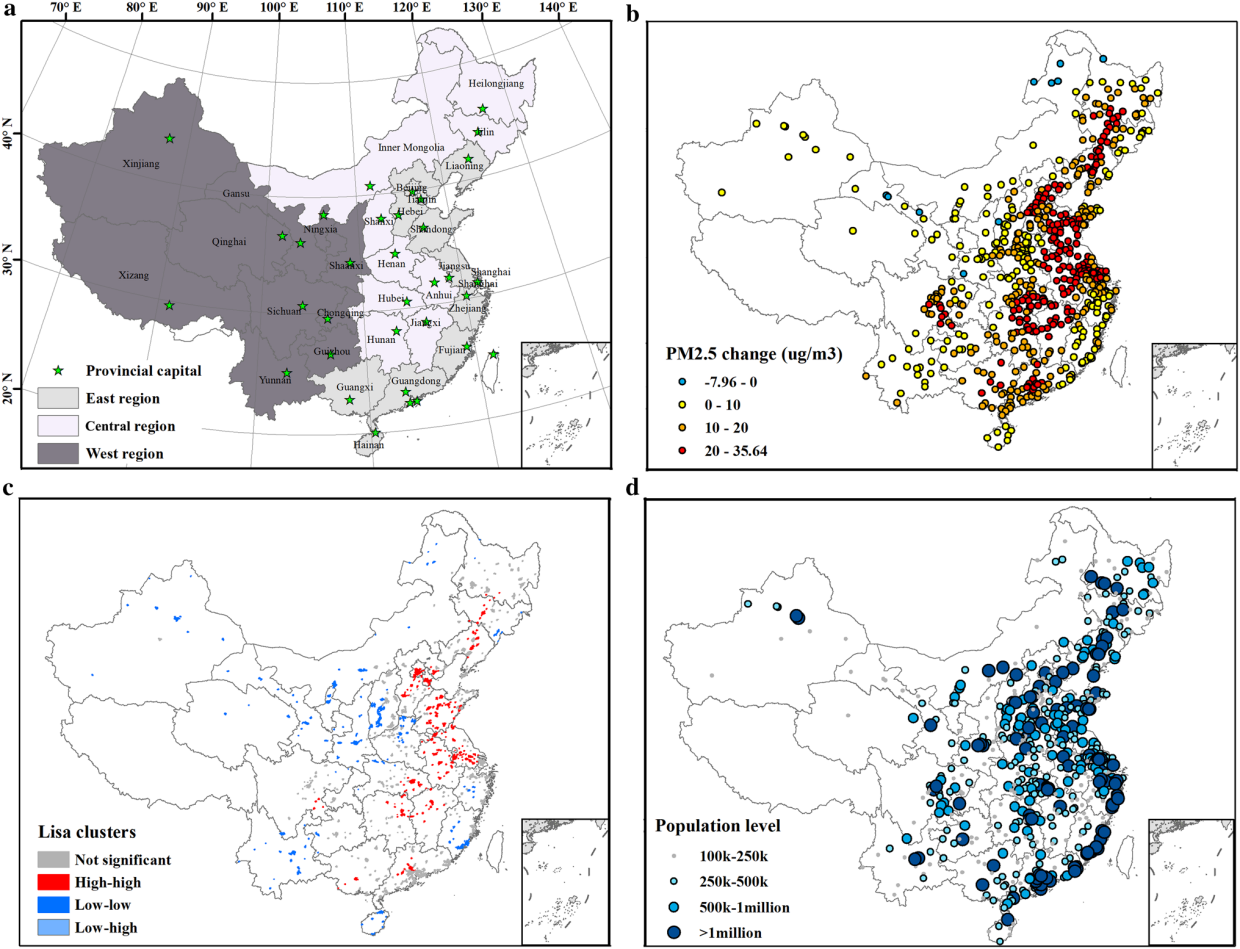
(**a**) Division of cities in China by socioeconomic development level and the locations of provincial capitals; (**b**) Changes in annual mean PM_2.5_ concentrations between the year 2000 and 2014; (**c**) LISA cluster maps for PM_2.5_ changes at the city level; High-high indicates a statistically significant cluster of high PM_2.5_ level changes over the study period. Low-low indicates a cluster of low PM_2.5_ inter-annual variation; No high-low cluster is reported; Low–high represents cities with high PM_2.5_ inter-annual variation surrounded by cities with low variation; (**d**) Population level by cities in the year 2014. Maps were produced by ArcGIS 10.7.1^85^.

**Figure 4:**
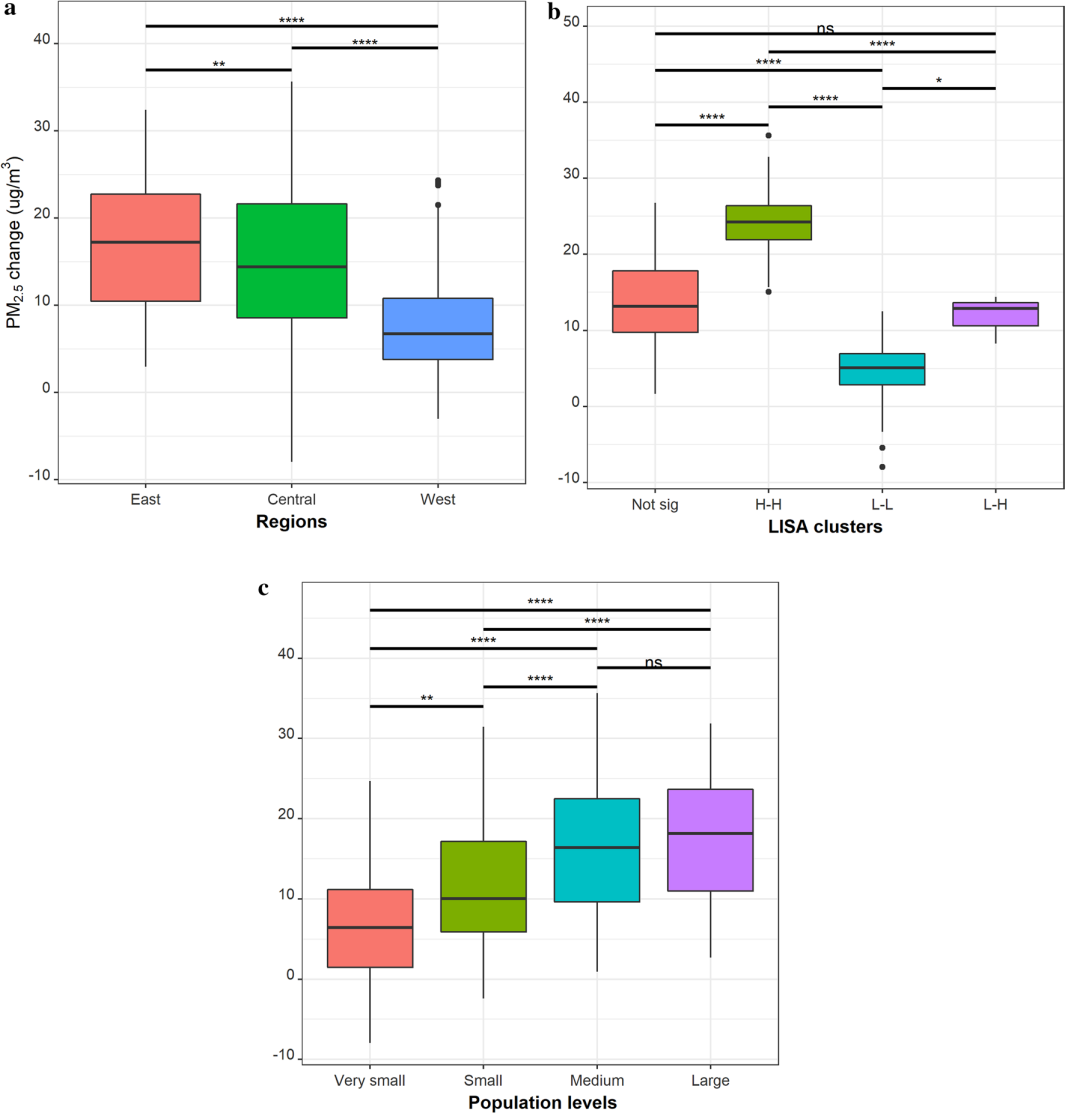
Boxplots of PM_2.5_ concentration changes between 2000 and 2014 for city groups that are formed according to (**a**) socioeconomic development level division, (**b**) LISA clusters, and (**c**) population level. Asterisk marks represent the *p* value of ANOVA significant test between the corresponding pair of groups. *Note* *ns* not significant; *p value < 0.05; **p value < 0.01; ***p value < 0.001; *H–H* high-high cluster, *L–H* low–high cluster, *L–L* denotes low–low cluster.

Spatially, the changes in PM_2.5_ levels exhibit heterogeneous patterns across cities (Fig. 3b). According to the socioeconomic level division (Fig. 3a), the Eastern, Central, and Western region experienced a 38.6, 35.3, and 25.5 µg/m^3^ increase in annual PM_2.5_ mean_,_ separately, and the difference among regions is significant according to the analysis of variance (ANOVA) results (Fig. 4a). When stratified by spatial autocorrelation relationship (Fig. 3c), the differences in PM_2.5_ changes among the spatial clusters are even more dramatic. The average PM_2.5_ increase in cities belonging to the high-high cluster is approximately 25 µg/m^3^, as compared to 5 µg/m^3^ in the low-low clusters (Fig. 4b). Finally, cities at four different population levels have significant differences in the changes of PM_2.5_ concentration (Fig. 3d), except for the mid-sized cities and large city agglomeration (Fig. 4c).

### The effects of urban forms on PM_2.5_ changes

The Hausman specification test for fixed versus random effects yields a *p* value less than 0.05, suggesting that the fixed effects model has better performance. We fit one panel data model to each city group and built nine models in total. All models are statistically significant at the *p* < 0.05 level and have moderate to high predictive power with the R^2^ values ranging from 0.63 to 0.95, which implies that 63–95% of the variation in the PM_2.5_ concentration changes can be explained by the explanatory variables (Table 2).

**Table 2:** Results of fix effected panel model.

	LISA Moran I		Socioeconomic development			Population (10 k people)			
	High-high	Low-low	Eastern	Central	Western	10–25	25–50	50–100	> 100
**Area metrics**									
TA	− 1786	− 5045	− 9064	2643	1038	− 5235	− 1150	604.42	2290
NP	1786	5045	9065	− 2643	− 1038	5236	1151	− 604.3	− 2290
PD	1696	− 2355	− 1932	1321	1102	− 4925	− 1122	292.85	20.14
AREA	3482	2699**	7132	− 1322	639**	3107	28.69	− 311.56	− 2270
**Shape metrics**									
SHAPE	0.87	1.24	4.76	4.75	19.54	− 36.04	2.57	1.11	1.89
FRAC	− 15.66	0.29	− 60.18*	− 31.35	− 119	224.75	− 20.29	− 12.24	− 32.64
CONTIG	0.67**	0.13	1.24**	− 0.43	0.29	− 2.89	0.43	0.64**	0.84**
**Aggregation metrics**									
LSI	10.02	− 0.05	− 0.83	0.07*	− 2.26	1.22	− 0.12	− 0.36	0.02
COHESION	− 5.8	− 0.92	− 8.50	− 7.38	6.36	10.85	2.97*	− 0.05	− 9.10
MESH	0.07	− 0.09	− 0.09	− 0.06	− 0.15	− 1.28	− 0.03	0.15	0.01
AI	− 0.62	0.45	− 3.58	3.78**	− 5.32	− 4.7	− 3.09*	− 1.50	2.79
**Control variables**									
POP	0.02	0.04	− 0.09	− 0.14	− 0.16	0.32	− 0.07	0.02	− 0.07
PopDen	0.03	0.05	− 0.07	− 0.20	− 0.05	0.40	− 0.05	0.03	− 0.09
GDP	0.00	0.00	− 0.04	0.15*	− 0.04	0.00	0.00	0.00*	0.001
GDP2	0.14***	0.04*	0.05	0.01	− 0.14	0.12	0.11***	0.12***	0.13***
TEMP	0.02	− 0.01	0.02**	0.02	− 0.002	− 0.04*	0.001	0.02***	0.01**
RH	1.45	− 0.39	1.12	2.81	0.58	− 0.09	0.63	2.26***	1.61*
WindSpeed	0.18***	0.1***	0.17***	0.06	0.12	0.34*	0.11***	0.11***	0.06
R^2^	0.95***	0.63***	0.90***	0.87***	0.83***	0.80*	0.76***	0.91***	0.87***

*Significance level of 0.05; **significance level of 0.01; ***significance level of 0.001.

The urban form—PM_2.5_ relationships differ distinctly in Eastern, Central, and Western China. All models reach high R^2^ values. Model for Eastern China (refer to hereafter as Eastern model) achieves the highest R^2^ (0.90), and the model for the Western China (refer to hereafter as Western model) reaches the lowest R^2^ (0.83). The shape metrics FRAC and CONTIG are correlated with PM_2.5_ changes in the Eastern model, whereas the area metrics AREA demonstrates a positive effect in the Western model. In contrast to the significant associations between shape, area metrics and PM_2.5_ level changes in both Eastern and Western models, no such association was detected in the Central model. Nonetheless, two aggregation metrics, LSI and AI, play positive roles in determining the PM_2.5_ trends in the Central model.

For models built upon the LISA clusters, the H–H model (R^2^ = 0.95) reaches a higher fitting degree than the L–L model (R^2^ = 0.63). The estimated coefficients vary substantially. In the H–H model, the coefficient of CONTIG is positive, which indicates that an increase in CONTIG would increase PM_2.5_ pollution. In contrast, no shape metrics but one area metrics AREA is significant in the L–L model.

The results of the regression models built for cities at different population levels exhibit a distinct pattern. No urban form metrics was identified to have a significant relationship with the PM_2.5_ level changes in groups of very small and mid-sized cities. For small size cities, the aggregation metrics COHESION was positively associated whereas AI was negatively related. For mid-sized cities and large agglomerations, CONTIG is the only significant variable that is positively related to PM_2.5_ level changes.

## Discussion

### Urban form is an effective measure of long-term PM_2.5_ trends

All panel data models are statistically significant regardless of the data group they are built on, suggesting that the associations between urban form and ambient PM_2.5_ level changes are discernible at all city levels. Importantly, these relationships are found to hold when controlling for population size and gross domestic product, implying that the urban landscape patterns have effects on long-term PM_2.5_ trends that are independent of regional economic performance. These findings echo with the local, regional, and global evidence of urban form effect on various air pollution types^5,14,21,22,24,39,78^ .

Although all models demonstrate moderate to high predictive power, the way how different urban form metrics respond to the dependent variable varies. Of all the metrics tested, shape metrics, especially CONTIG has the strongest effect on PM_2.5_ trends in cities belonging to the high-high cluster, Eastern, and large urban agglomerations. All those regions have a strong economy and higher population density^86^. In the group of cities that are moderately developed, such as the Central region, as well as small- and mid-sized cities, aggregation metrics play a dominant negative role in PM_2.5_ level changes. In contrast, in the least developed cities belonging to the low-low cluster regions and Western China, the metrics describing size and number of urban patches are the strongest predictors. AREA and NP are positively related whereas TA is negatively associated.

### The impacts of urban form metrics on air quality vary by urbanization degree

**Figure 5:**
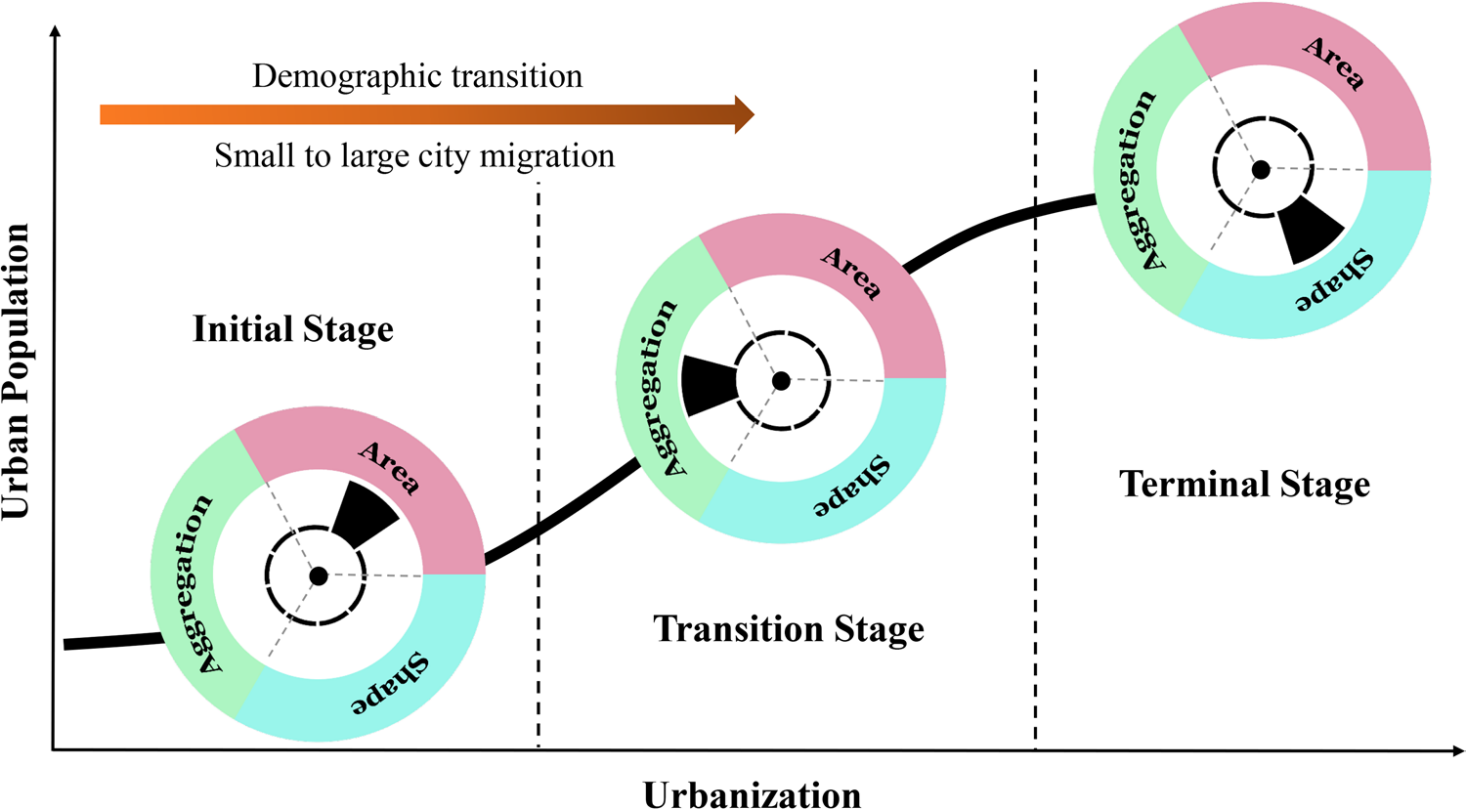
The most influential metric of urban form in affecting PM_2.5_ level changes at different urbanization stages.

Based on the above observations, how urban form affects within-city PM_2.5_ level changes may differ over the urbanization stages. We conceptually summarized the pattern in Fig. 5: area metrics have the most substantial influence on air pollution changes at the early urban development stage, and aggregation metrics emerge at the transition stage, whereas shape metrics affect the air quality trends at the terminal stage. The relationship between urban form and air pollution has rarely been explored with such a wide range of city selections. Most prior studies were focused on large urban agglomeration areas, and thus their conclusions are not representative towards small cities at the early or transition stage of urbanization.

Not surprisingly, the area metrics, which describe spatial grain of the landscape, exert a significant effect on PM_2.5_ level changes in small-sized cities. This could be explained by the unusual urbanization speed of small-sized cities in the Chinese context. Their thriving mostly benefited from the urbanization policy in the 1980s, which emphasized industrialization of rural, small- and mid-sized cities^87^. With the large rural-to-urban migration and growing public interest in investing real estate market, a side effect is that the massive housing construction that sometimes exceeds market demand. Residential activities decline in newly built areas of smaller cities in China, leading to what are known as ghost cities^88^. Although ghost cities do not exist for all cities, high rate of unoccupied dwellings is commonly seen in cities under the prefectural level. This partly explained the negative impacts of TA on PM_2.5_ level changes, as an expanded while unoccupied or non-industrialized urban zones may lower the average PM_2.5_ concentration within the city boundary, but it doesn’t necessarily mean that the air quality got improved in the city cores.

Aggregation metrics at the landscape scale is often referred to as landscape texture that quantifies the tendency of patch types to be spatially aggregated; i.e., broadly speaking, aggregated or “contagious” distributions. This group of metrics is most effective in capturing the PM_2.5_ trends in mid-sized cities (population range 25–50 k) and Central China, where the urbanization process is still undergoing. The three significant variables that reflect the spatial property of dispersion, separately landscape shape index, patch cohesion index, and aggregation index, consistently indicate that more aggregated landscape results in a higher degree of PM_2.5_ level changes. Theoretically, the more compact urban form typically leads to less auto dependence and heavier reliance on the usage of public transit and walking, which contributes to air pollution mitigation^89^. This phenomenon has also been observed in China, as the vehicle-use intensity (kilometers traveled per vehicle per year, VKT) has been declining over recent years^90^. However, VKT only represents the travel intensity of one car and does not reflect the total distance traveled that cumulatively contribute to the local pollution. It should be noted that the private light-duty vehicle ownership in China has increased exponentially and is forecast to reach 23–42 million by 2050, with the share of new-growth purchases representing 16–28%^90^. In this case, considering the increased total distance traveled, the less dispersed urban form can exert negative effects on air quality by concentrating vehicle pollution emissions in a limited space.

**Figure 6:**
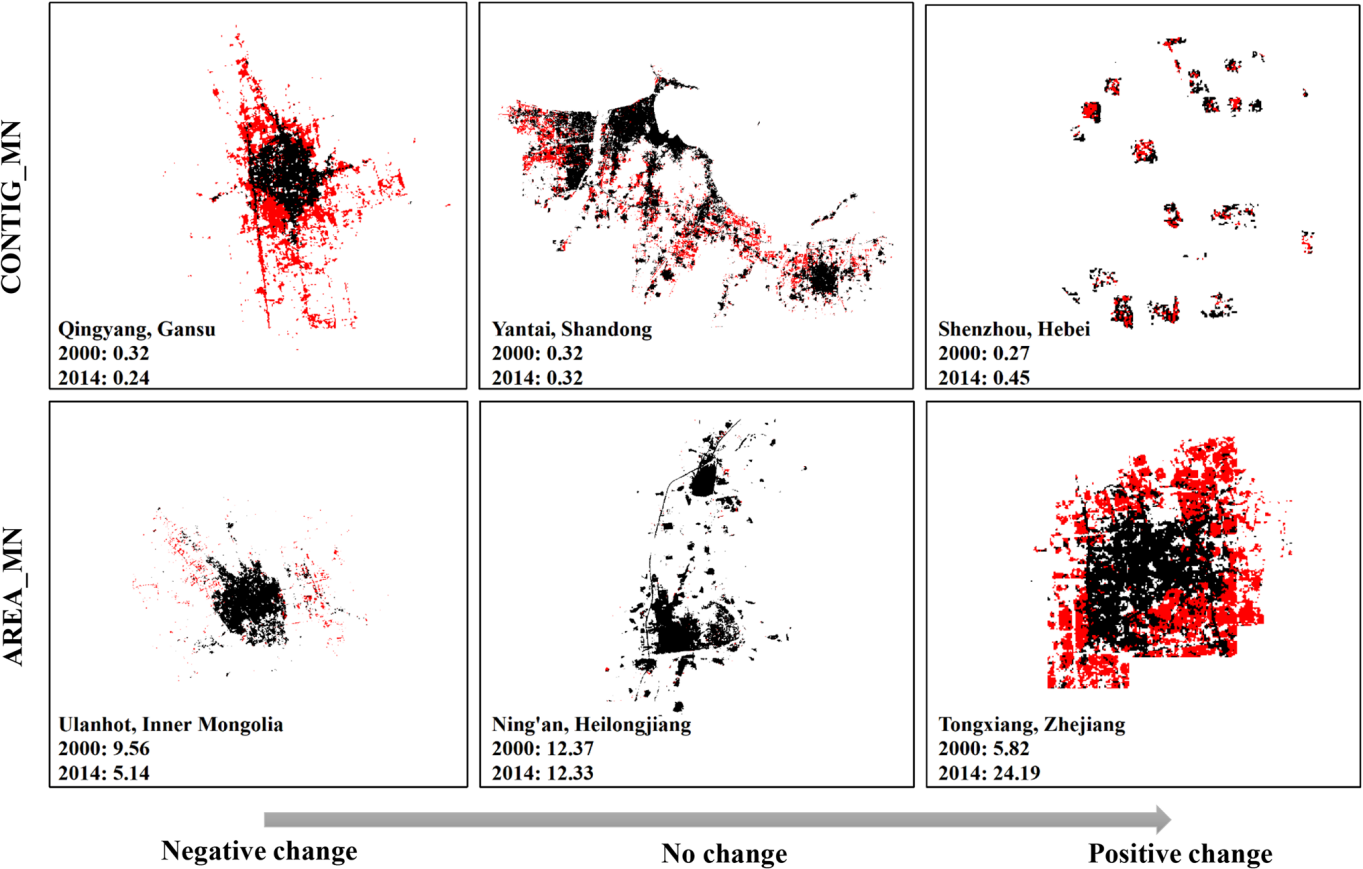
Six cities illustrating negative to positive changes in CONTIG_MN and AREA_MN. Pixels in black show the urban areas in the year 2000 and pixels in red are the expanded urban areas from the year 2000 to 2014. Figure was produced by ArcGIS 10.7.1^85^.

Finally, urban contiguity, observed as the most effective shape metric in indicating PM_2.5_ level changes, provides an assessment of spatial connectedness across all urban patches. Urban contiguity is found to have a positive effect on the long-term PM_2.5_ pollution changes in large cities. Urban contiguity reflects to which degree the urban landscape is fragmented. Large contiguous patches result in large CONTIG_MN values. Among the 626 cities, only 11% of cities experience negative changes in urban contiguity. For example, Qingyang, Gansu is one of the cities-featuring leapfrogs and scattered development separated by vacant land that may later be filled in as the development continues (Fig. 6). Most Chinese cities experienced increased urban contiguity, with less fragmented and compacted landscape. A typical example is Shenzhou, Hebei, where CONTIG_MN rose from 0.27 to 0.45 within the 14 years. Although the 13 counties in Shenzhou are very far scattered from each other, each county is growing intensively internally rather than sprawling further outside. And its urban layout is thus more compact (Fig. 6). The positive association revealed in this study contradicts a global study indicating that cities with highly contiguous built-up areas have lower NO_2_ pollution^22^. We noticed that the principal emission sources of NO_2_ differ from that of PM_2.5._ NO_2_ is primarily emitted with the combustion of fossil fuels (e.g., industrial processes and power generation)^6^, whereas road traffic attributes more to PM_2.5_ emissions. Highly connected urban form is likely to cause traffic congestion and trap pollution inside the street canyon, which accumulates higher PM_2.5_ concentration. Computer simulation results also indicate that more compact cities improve urban air quality but are under the premise that mixed land use should be presented^18^. With more connected impervious surfaces, it is merely impossible to expect increasing urban green spaces. If compact urban development does not contribute to a rising proportion of green areas, then such a development does not help mitigating air pollution^41^.

## Conclusions

This study explores the regional land-use patterns and air quality in a country with an extraordinarily heterogeneous urbanization pattern. Our study is the first of its kind in investigating such a wide range selection of cities ranging from small-sized ones to large metropolitan areas spanning a long time frame, to gain a comprehensive insight into the varying effects of urban form on air quality trends. And the primary insight yielded from this study is the validation of the hypothesis that the determinants of PM_2.5_ level trends are not the same for cities at various developmental levels or in different geographic regions. Certain measures of urban form are robust predictors of air quality trends for a certain group of cities. Therefore, any planning strategy aimed at reducing air pollution should consider its current development status and based upon which, design its future plan. To this end, it is also important to emphasize the main shortcoming of this analysis, which is generally centered around the selection of control variables. This is largely constrained by the available information from the City Statistical Yearbook. It will be beneficial to further polish this study by including other important controlling factors, such as vehicle possession.
